# Validation of a freely distributable software for the analysis of electrophysiological signals: Smart Tools for Evoked Potentials (STEP)

**DOI:** 10.1590/2317-1782/e20240265en

**Published:** 2025-08-08

**Authors:** Kelly Cristina Lira de Andrade, Aline Tenório Lins Carnaúba, Carlos Henrique Alves Batista, Danielle Cavalcante Ferreira, Raí Fernandes Santos, Raquel da Silva Cabral, Pedro de Lemos Menezes

**Affiliations:** 1 Programa Associado de Pós-graduação em Fonoaudiologia – PPGFON (UFPB/UFRN/UNCISAL), Universidade Estadual de Ciências da Saúde de Alagoas – UNCISAL - Maceió (AL), Brasil.; 2 Laboratório de Computação Científica e Análise Numérica, Universidade Federal de Alagoas – UFAL - Maceió (AL), Brasil.

**Keywords:** Audiology, Evoked Potentials, Evoked Potentials, Auditory, Validation Study, Computer-Assisted Signal Processing

## Abstract

**Purpose:**

This study aimed to validate the STEP, an application developed for the analysis of various auditory and vestibular electrophysiological signals. The STEP was designed to enhance the accuracy and efficiency of latency and amplitude analysis, as well as other waveform morphological features such as calculation of area, slope, and Fast Fourier Transform (FFT).

**Methods:**

The methodology was structured into two phases: one involving simulated waveforms and the other based on experimental data. In the first phase, waveforms were generated using mathematical functions, and their features were marked and analyzed both by trained examiners and by the STEP application. In the second phase, the STEP was tested using real electrophysiological recordings, with latency and amplitude values compared across STEP and two established gold-standard systems.

**Results:**

The results demonstrated high accuracy of STEP in both manual and automatic peak and trough markings, as well as in subsequent calculations. No statistically significant differences were found among the evaluated systems, nor between the examiners.

**Conclusion:**

The STEP proved to be a reliable tool for identifying latencies and amplitudes of electrophysiological waveforms and for performing additional analyses, including P1N1 area calculation, slope estimation, and FFT analysis.

## INTRODUCTION

Recently, advances in information technology have enabled the development of new tools aimed at improving the efficiency and accuracy of clinical analyses. Several studies have highlighted the importance of validating these emerging technologies prior to their implementation in research settings and clinical practice^(1,2)^. For instance, one study has observed the use of artificial intelligence through a cardiac signal analysis algorithm, demonstrating the importance of such comparisons to ensure reliability in new methods^(3)^. Conversely, another study has validated an electroencephalogram (EEG) analysis software, comparing its results with those of a widely used gold standard system^(4)^. Additionally, the validation of an auditory electrophysiological response analysis software has highlighted the need for accurate tools for clinical practice in Audiology^(5)^. Nevertheless, only few initiatives for more in-depth analysis of auditory electrophysiological waves go beyond the simple marking of latencies, amplitudes and interpeak intervals.

In this context, electrophysiological assessments are highlighted, more specifically the BAEP (Brainstem Auditory Evoked Potentials), in which the accurate identification of wave latencies and amplitudes is crucial for the correct analysis of results and appropriate clinical referrals. However, conventional measurements of latency and amplitude may be insufficient to capture the full complexity of electrophysiological waveforms. Studies have emphasized the importance of more detailed morphological analyses, such as the calculation of the area under the curve, slope, and Fast Fourier Transform, which enable a deeper understanding of auditory and neurological functioning, enhancing accuracy of exam reports and clinical conclusions^(6,7)^.

The literature also includes a study that has observed small variations in latency and amplitude in electrophysiological assessments conducted with equipment from different manufacturers^(8)^. In the supplementary material of one of the studies, for instance, the researchers simultaneously recorded a single exam with three different systems (IHS, Bio-logic Navigator Pro and Compumedics Neuroscan System). Despite using the same patients, protocol, electrode montage and environmental conditions, variations in the latencies and amplitudes of the frequency following response (FFR) were observed, particularly in components A, C and D^(8)^. These findings indicate that, although wave morphologies are apparently similar, the specific technical characteristics of each equipment, such as their algorithms and hardware, may impact the findings.

Given this scenario, this study proposes the validation of the *Smart Tools for Evoked Potentials* (STEP), a software developed by a public university in Brazil and designed to increase the morphological analysis capabilities of all auditory and vestibular electrophysiological tests frequently used in auditory assessment. The application provides greater accuracy and efficiency in the analysis of wave latencies and amplitudes, and in conducting other morphological analyses, such as calculation of the area, slope, and Fast Fourier Transform. This investigation is expected to generate robust evidence supporting the adoption of the STEP application as a valid and effective tool for healthcare professionals, especially those in the field of Audiology.

## METHOD

### Smart Tools Evoked Potentials (STEP)

The application comprises three main tools designed to support, respectively, the teaching of electrophysiology and the training of professionals; the marking and analysis of responses from any electrophysiological potential recorded during auditory assessments; and the development of research.

The first tool consists of a system capable of simulating electrophysiological waves, studying the repercussions of changes in each protocol parameter on the waveforms, such as filters, stimulation rate, types of stimuli, etc.

The second function, object of this study, enables the upload of electrophysiological recordings from any patient, displays the data on a graph, and allows marking the peaks and troughs of the electrophysiological waves, self-marking these peaks and troughs, calculating latencies, amplitudes, inter-latency and inter-amplitude intervals, calculating the slope and the area between two points using several techniques, calculating angles, area ratio, Mismatch Negativity (MMN), calculating asymmetry indices, and the binaural interaction component (BIC). In addition, this function supports the application of the Fast Fourier Transform of the entire time window or a selected time interval, and both manual and automatic marking of amplitude peaks for each frequency.

The third function, used to conduct research, facilitates the tabulation of data in Excel spreadsheets, tests research protocols, and offers support for carrying out statistical analyses.

The application was developed in the C++ programming language, with the assistance of the QT 6.3 tool, for macOS 14.5. A version for Windows was subsequently developed. STEP is currently in version 2.6 for both operating systems.

### Study methodology

This study aims to validate some of the measurements performed by the STEP application, establishing its accuracy for such processing. Furthermore, one of the motivations for developing STEP was to democratize access to advanced clinical analysis technologies in the area. Therefore, the application will be made available free of charge to other public institutions, including universities, hospitals, research institutes, among others. The methodology of this study prioritized a rigorous methodological approach, including theoretical, experimental, and practical comparisons. Validation procedures were performed for both time-domain and frequency-domain measures. In the time domain, the following elements were validated: (i) manual and automatic marking of latencies and amplitudes; (ii) calculation of the area under the curve between two points using integral methods and the summation of two triangles; and (iii) the calculation of the slope. In the frequency domain, the validation encompassed: (i) the marking of frequency peaks and (ii) their corresponding amplitude values, both manually and automatically.

**Figure 1:**
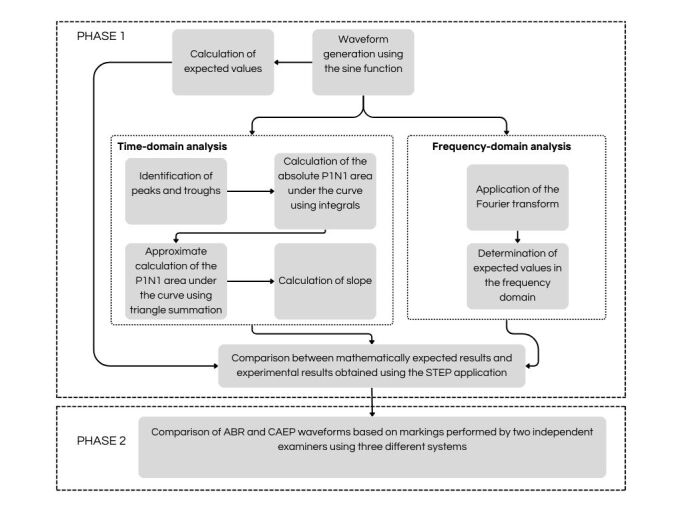
Schematic summary of the methodology

The methodology framework of the study is organized into two primary phases, which are illustrated in Figure 1 and detailed below.

**Phase I.** Compare the obtained results, using the STEP application, with the expected results, through mathematical calculations^^1^^.

**1. Wave generation:** Initially, three waves were generated in Python version 3.10.1 for macOS 14.5 (Appendix I) and saved in Microsoft Excel format; three waveforms from sine functions and a fourth waveform that represented the sum of the three. The sine waves in the time domain were generated using the equation:


$$y\left(t\right)\;=\;A\;sin\left(2\;\pi \;f\;t\right)$$ (1)

where:

$y\left(t\right)$ represents the value of the wave generated at a given time t,

A is the amplitude, indicating the maximum height the wave reaches relative to a reference baseline,

sin is the trigonometric sine function that describes the periodic oscillation of the wave,

$2\pi \;\left(rad\right)$ corresponds to a full cycle in the trigonometric circle,

f is the frequency of the wave, i.e., the number of complete cycles the wave completes in 1 second.

The parameters used were: frequencies of 0.5 kHz, 1 kHz and 2 kHz; time interval ranging from  0 ms a 10 ms; peak amplitude: 10 μV; trough amplitude: -10 μV; peak-to-trough amplitude: $10-\left(-10\right)=20\;\mu V$; A total of 3072 Cartesian coordinates $\left(x,\;y\right)$ were generated for each waveform. Accordingly, the following data were stored: time progression $\left(t\right)$, ranging from 0 a 10 ms; amplitudes for $0.5\;kHz\;\left(y1\right)$; amplitudes for $1\;kHz\;\left(y2\right)$; amplitudes for $2\;kHz\;\left(y3\right)$; and the sum of the three waveforms $\left(y4\;=\;y1+y2+y3\right)$.

## CALCULATION OF EXPECTED VALUES IN TIME DOMAIN

### Mathematical functions used

#### Mathematical identification of Peaks and Troughs

- For a sinusoidal wave y( *t* ) = *A sin( 2 π ft)*, the first peaks and troughs occur under the following conditions: peak: $t_{peak\;1}=\;\frac{1}{4f}$; trough: $t_{trough\;1}=\;\frac{3}{4f}\;$; zero-cross: $t_{zero-cross}=\;\frac{1}{2f}\;$;

#### Calculation of the absolute area P1N1 under the curve

The area under the curve represents the total sum of the values under a curve on a graph. Integral equations can be used to calculate this area. This measurement is useful for understanding the magnitude of phenomena, such as the total amount of a signal over time.

The area $A_{area}$ below the curve between the first peak and the first trough can be calculated by the integral of the sine function between $t_{peak\;}$and $t_{trough\;}$:


$$A_{area}=\;\overset{t_{trough\;}}{\underset{t_{peak\;}}{\int }}A\;sin\left(2\pi ft\right)\;dt$$ (2)

Using the trigonometric identities and the integral of the sine function:


$$A_{area}=-\frac{A}{2\pi f}cos(2\pi ft)\left\{\begin{array}{c} t_{trough\;}\\ t_{peak\;} \end{array}\right.$$ (3)

Substituting the values $t_{peak\;}$and $t_{trough\;}$:


$$A_{area}=-\frac{A}{2\pi f}[cos[2\pi ft_{trough\;})-\left(2\pi ft_{peak\;}\right)]$$ (4)

#### Calculation of the area P1N1 under the curve, approximated by two triangles

The area can also be calculated approximately by adding the area of two triangles.


$$A_{area\;two\;triangles}=\;A_{area\;triangle\;1}+\;A_{area\;triangle\;2}$$ (5)

$$A_{area\;triangle\;1}=\;\frac{\begin{array}{l} \left(time\;interval\;between\;peak\;and\;zero\;(\text{crossing point}\right))\times \\ \;\left(peak\;amplitude\right) \end{array}}{2}$$ (6)

$$A_{area\;triangle\;2}=\;\frac{\begin{array}{l} \left(time\;interval\;between\;\text{zero}\left(\text{crossing point}\right)\text{and the trough}\right)\times \;\\ \left(trough\;amplitude\right) \end{array}}{2}$$ (7)

#### Slope calculation

The slope is a measure of how quickly one variable changes in relation to another. On a straight line on a graph, the slope is calculated as the difference in height (y) divided by the difference in base (t). The formula is:


$$Slope=\frac{\Delta y}{\Delta t}=\frac{variation\;of\;amplitude\;between\;peak\;and\;trough}{Time\;interval\;between\;peak\;and\;trough}$$ (8)

### Values found for each of the frequencies

Calculation of t and y(t) values for f equal to 0.5 kHz, 1 kHz, and 2 kHz, considering an amplitude range from –10 to 10 microvolts, for peak, trough, and zero-cross intervals.

#### Frequency of 0.5 kHz


$$peak:t=\frac{1}{4f}=\frac{1}{4X500}=\frac{1}{2000}=0.0005\;s$$ (9)

$$peak:t_{peak\;1}=0.5\;ms;$$ (10)

$$\begin{array}{l} y\left(t\right)=Asin\left(2\pi ft\right)=y\left(0.0005\right)=10sin(2\pi 500x0.0005)=\\ 10sin(2\pi 0.25)=10sin(\frac{\pi }{2})=10\;\mu V \end{array}$$ (11)

$$Trough:t=\frac{3}{4f}=\frac{3}{4X500}=\frac{3}{2000}=0.0015\;s$$ (12)

$$Trough:t_{\;trough\;1}=\;1.5\;ms$$; (13)

$$\begin{array}{l} y\left(t\right)=Asin\left(2\pi ft\right)=y\left(0.0015\right)=10sin(2\pi 500x0.0015)=\\ 10sin(2\pi 0.75)=10sin(\frac{3\pi }{2})=-10\;\mu V \end{array}$$ (14)

$$Zero-cross:t=\frac{1}{2f}=\frac{1}{2X500}=\frac{1}{1000}=0.001\;s$$ (15)

$$Zero-cross:t_{P1N1}=1\;ms.$$ (16)

$$\begin{array}{l} y\left(t\right)=Asin\left(2\pi ft\right)=y\left(0.001\right)=10sin(2\pi 500X0.001)=\\ 10sin(2\pi 0.5)=10sin(\pi )=\;0\;\mu V \end{array}$$ (17)

Absolute area under the curve*:*$A_{area\;1}=\;\overset{1.5\;ms}{\underset{0.5\;ms}{\int }}10\;sin\left(2\pi 500t\right)\;dt=6.36$ μVms*.* Area P1N1 below the curve, approximated by two triangles:


$$A_{area\;triangle\;1}=\;\frac{\left(1-0.5\right)\times \;\left(10\right)}{2}=2.5\;\mu Vms$$ (18)

$$A_{area\;triangle\;2}=\;\frac{\left(1.5-1\right)\times \;\left(10\right)}{2}=2.5\;\mu Vms$$ (19)

$$A_{area\;two\;triangles}=5\;\mu Vms$$ (20)

$$Slope:Slope_{0.5kHz}=\frac{20}{1}=20\mu V/ms$$ (21)

#### Frequency of 1 kHz

Peaks: peak: $t_{peak\;1}=0.25\;ms;$ trough: $t_{trough\;1}=\;0.75\;ms$*;* zero-cross: $t_{P1N1}=0.50\;ms;$

Absolute area under the curve:$A_{area\;1}=\;\overset{0.75\;ms}{\underset{0.25\;ms}{\int }}10\;sin\left(2\pi 1000t\right)\;dt=3.18$ μVms*;* Area P1N1 below the curve, approximated by two triangles:


$$A_{area\;triangle\;1}=\;\frac{\left(0.50-0.25\right)\times \;\left(10\right)}{2}=1.25\;\mu Vms$$ (22)

$$A_{area\;triangle\;2}=\;\frac{\left(0.75-0.50\right)\times \;\left(10\right)}{2}=1.25\;\mu Vms$$ (23)

$$A_{area\;two\;triangles}=2.5\;\mu Vms$$ (24)

$$Slope:Slope_{1kHz}=\frac{20}{0.5}=40\mu V/ms$$ (25)

#### Frequency of 2 kHz

Peaks: peak: $t_{peak\;1}=0.125\;ms;$ trough: $t_{trough\;1}=\;0.375\;ms$*;* zero-cross: $t_{P1N1}=0.25\;ms;$

Absolute area under the curve: $A_{area\;1}=\;\overset{0.375\;ms}{\underset{0.125\;ms}{\int }}10\;sin\left(2\pi 2000t\right)\;dt=1.59$ μVms*;* Area P1N1 below the curve, approximated by two triangles:


$$A_{area\;triangle\;1}=\;\frac{\left(0.25-0.125\right)\times \;\left(10\right)}{2}=0.625\;\mu Vms$$ (26)

$$A_{area\;triangle\;2}=\;\frac{\left(0.375-0.25\right)\times \;\left(10\right)}{2}=0.625\;\mu Vms$$ (27)

$$A_{area\;two\;triangles}=1.25\;\mu Vms$$ (28)

$$Slope:Slope_{2kHz}=\frac{20}{0.25}=80\mu V/ms$$ (29)

## CALCULATION OF EXPECTED VALUES IN THE FREQUENCY DOMAIN

Frequency-domain analysis involves transforming signals from the time domain to the frequency domain, enabling the identification of the frequency components present in a composite signal. To perform this analysis, the Fourier Transform was utilized, as it decomposes time-domain function into its sinusoidal components across different frequencies.

### Fourier Transform

The Fourier Transform is a mathematical tool that transforms a continuous signal in time-domain into a continuous signal in frequency domain. The Fourier Transform formula for a function $y\left(t\right)$is given by:


$$Y\left(f\right)=\overset{\infty }{\underset{-\infty }{\int }}y\left(t\right)e^{-j2\pi ft}dt$$ (30)

where:

$Y\left(f\right)$ is the Fourier Transform of $y\left(t\right)$,

j is the imaginary unit$\left(\sqrt{-1}\right)$

f is the frequency,

t is the time.

For discrete signals, as in the case of the data generated for this experiment, the Discrete Fourier transform (DFT) is used, computed through the Fast Fourier Transform (FFT) algorithm.

#### Application of Fourier Transform

To analyze the frequencies present in the sum signal $y_{4}\left(t\right)$ (result of the sum of the three sine waves), we have applied the FFT to the signal data. The FFT transforms the signal from the time domain to the frequency domain, revealing the frequency components and their amplitudes. Considering that the signal $y_{4}\left(t\right)$ is a sum of three sine waves with frequencies of 0.5 kHz, 1 kHz and 2 kHz, and that each of these waves has a peak-to-peak amplitude of 20 μV, amplitude peaks in the frequency spectrum corresponding to these frequencies are expected.

### Values found in the frequency domain

After applying the FFT, the results in the frequency domain are represented in a graph where the horizontal axis (x) represents the frequency, and the vertical axis (y) represents the corresponding amplitude.

Thus, after applying the FFT, we expect to find a peak in each of the separate waves (0.5 kHz, 1 kHz and 2 kHz), at exactly the same frequencies, and the three peaks together in the composite waveform (y_4_). All expected, standardized peaks should have 10 μV of amplitude, as seen below. These peaks were marked by two experienced examiners, and automatically, using one of the functions available in STEP. For a sinusoidal wave $y\left(t\right)=A\;\text{sin}\left(2\pi ft\right)$, where *A* is the peak amplitude and *f* is the frequency. The amplitude observed in the frequency spectrum after the FFT is:


$$A_{freq}=\;\frac{A_{peak-to-peak}}{2}$$ (31)

where:


$$A_{peak-to-peak}=20\;\mu V\;\left(10\;\mu V\;to-10\;\mu V\right)$$ (32)

Thus, the expected amplitude in the frequency domain for the 1 kHz component is:


$$A_{freq}=\;\frac{20\;}{2}=10\;\mu V$$ (33)

Therefore, after applying the FFT, the amplitude of the component of each frequency is expected to be 10 μV.

## COMPARISON BETWEEN THE MATHEMATICALLY EXPECTED RESULTS AND EXPERIMENTAL RESULTS OBTAINED USING THE STEP APPLICATION

Two trained examiners manually marked the peaks and troughs of the generated waveforms using the STEP application. In addition to the examiners' measurements, a third measurement was obtained using the application’s automatic marking function, which was considered the third examiner. After marking the peaks, the application's “calculate” function was used to automatically calculate latencies, amplitudes, interpeak intervals, areas, slopes, and other parameters. This process was repeated for each of the three confirmed sine waves. Subsequently, the application's FFT function was applied to each of the three individual frequencies and for their sum.

To compare the theoretical data with the data marked by the examiners and automatically by STEP (third examiner), the following metrics were used:

The mean absolute deviation (MAD) was employed to quantify the mean absolute differences between the theoretical peak values and those obtained via the STEP application. This metric is particularly useful for assessing the accuracy of estimated values. As it relies on absolute differences, MAD is robust to outliers and provides a reliable indication of how closely the observed measurements approximate the expected theoretical values, regardless of the presence of outliers;
Mean absolute percentage error (MAPE), in turn, was used to measure the relative accuracy of measurements by calculating the mean of the absolute percentage differences between estimated and theoretical values. This metric is widely used as it expresses the error in percentage terms, facilitating interpretation and comparison across different data sets. MAPE provides a direct view of how close, in percentage terms, measurements are to expected values, and is especially useful for assessing the effectiveness of models or measurement tools;
Pearson's correlation coefficient was utilized to assess the correlation between theoretical values and values obtained with the application;
The intraclass correlation coefficient (ICC) to assess intra-examiner and inter-examiner agreement.

**Phase II.** Comparison of electrophysiological tracings, brainstem auditory evoked potentials (BAEP), and cortical auditory evoked potentials (CAEP), marked by two experienced examiners in the clinical gold standard system (AEP), in the gold standard system for mathematical calculations (Microsoft Excel), and in the STEP.

To prove the effectiveness of the measurements performed by the STEP, it would be sufficient to prove its accuracy with mathematically controlled waves, as performed in phase I. However, for an effective evaluation of real electrophysiological data, and as additional proof, we decided to evaluate the application in the analysis of the BAEP, as it is the most widely used short-latency potential in auditory electrophysiological evaluations, and the CAEP, as it is one of the most widely used long-latency potentials in the area. The aim of this phase was to compare electrophysiological recordings using the AEP application (version 7.3) from the Biologic Pro Navigator system. These recordings were subsequently converted into comma-separated values (CSV) text files with the assistance of the ASCII application (version 2.0) provided by Biologic. The system was considered the gold standard and is widely adopted in clinical audiology practice worldwide. The waveforms were marked by two experienced clinical examiners directly within the software used for data acquisition (AEP Biologic), in Microsoft Excel, and in the analysis application (STEP). The resulting data were subsequently compared using statistical techniques.

Initially, 15 assessments were selected by simple random sampling among the electrophysiological tests in the database of a public university. Information from the waveform recordings was extracted, in addition to other secondary information: patient's sex, age, and condition, without hearing impairment, only to confirm the inclusion criteria of the tests to be assessed. These waveforms were exported to text format using the platform of the clinical equipment used. Subsequently, the files were imported into Microsoft Excel and STEP. The marking was performed at different and random times. Thus, two experienced clinical examiners manually marked the peaks and troughs of the waves in the three applications, in estimated order, recording their latencies and amplitudes. Finally, the mean, standard deviation, and 95% confidence intervals of the latencies and amplitudes marked by both examiners in each application were calculated. The repeated-measures ANOVA was applied to each variable in both the BAEP (latencies and amplitudes of waves I, III, and V) and in the CAEP (latencies and amplitudes of waves P1, N1, and P2) to determine whether there were significant differences among the waveforms marked by the different systems (AEP, *Microsoft* Excel*,*and *STEP).* In addition, the Student's t test was applied for paired measures, with Bonferroni correction, to observe whether there were significant differences between examiners 1 and 2. The value was used to observe the magnitude of the difference observed between them.

Finally, the area measurements using the two tested methods, integral below the curve and sum of the two triangles, were compared using the Wilcoxon test, and the effect sizes were calculated using Cohen's d test.

The comparison across the three evaluated systems was performed using the ANOVA, with the F value representing the ratio between the variation among groups and the variation within groups.

All statistical tests were conducted using IBM SPSS version 28 for macOS Sonoma 14.5. Sample normality was calculated using the Shapiro-Wilk test and sphericity using the Mauchly test. For non-spherical factors, the p-value was obtained using the Greenhouse-Geisser test. Values were considered significant when p < 0.05. The established beta value was 0.1.

## RESULTS

### Phase I

The expected results, mathematically, for the latencies and amplitudes of the peaks and troughs, zero-time crosses, P1N1 areas below the curve, area of the sum of the two triangles, and slope found, by frequency, are shown in Table 1. Table 1 also includes the values of these measurements found experimentally, with STEP, for each of the examiners and for the automatic marking of STEP (examiner 3).

**Table 1:** Expected values and values obtained by examiners using the STEP, for each measurement and generated frequency, in the time domain

Measures	Expected	Examiner 1	Examiner 2	Examiner 3*
500 Hz peak 1: Latency (ms)	0.5	0.5	0.5	0.5
1000 Hz peak 1: Latency (ms)	0.25	0.25	0.25	0.25
2000 Hz peak 1: Latency (ms)	0.12	0.12	0.12	0.12
All frequencies peak 1: Amplitude (µV)	10	10	10	10
500 Hz trough 1: Latency (ms)	1.5	1.5	1.5	1.5
1000 Hz trough 1: Latency (ms)	0.75	0.74	0.74	0.75
2000 Hz trough 1: Latency (ms)	0.37	0.37	0.37	0.37
All frequencies are worth 1: Amplitude (µV)	-10	-10	-10	-10
500 Hz: zero-cross (ms)	1	1	1	1
1000 Hz: zero-cross (ms)	0.5	0.5	0.5	0.5
2000 Hz: zero-cross (ms)	0.25	0.25	0.25	0.25
500 Hz: Area under the curve (µV*ms)	6.36	6.33	6.33	6.33
1000 Hz: Area under the curve (µV*ms)	3.18	3.05	3.05	3.15
2000 Hz: Area under the curve (µV*ms)	1.59	1.56	1.56	1.56
500 Hz: Area of two triangles (µV*ms)	5	5	5	5
1000 Hz: Area of two triangles (µV*ms)	2.5	2.5	2.5	2.5
2000 Hz: Area of two triangles (µV*ms)	1.25	1.25	1.25	1.25
500 Hz: Slope (µV/ms)	20	20	20	20
1000 Hz: Slope (µV/ms)	40	40.81	40.81	40
2000 Hz: Slope (µV/ms)	80	80	80	80

* Examiner 3: automatic STEP marking

**Caption:** Hz – hertz; ms – milliseconds; µV – microvolts

**Figure 2:**
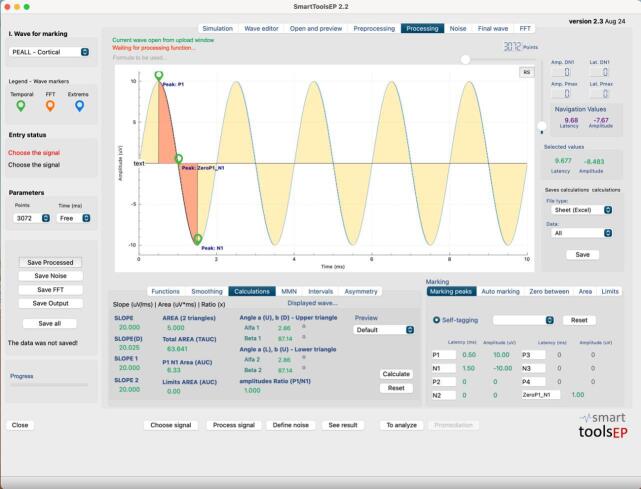
Peak and trough marking screen of the STEP application. Example of a marked waveform for the 0.5 kHz frequency

Figure 2 displays an example of the waveforms marked with the STEP application, for the frequency of 0.5 kHz.

Analysis of the waveform markings, peaks and troughs, and their respective latencies and amplitudes, reveals that the human examiners, identified as Examiner 1 and Examiner 2, presented a mean absolute difference (MAD) of 0.0009, indicating a very small mean difference in relation to the expected values. The mean absolute percentage error (MAPE) for both was 0.0012%, reflecting a high accuracy in the measurements, although not absolutely perfect. In contrast, Examiner 3, representing the STEP automatic marking system, demonstrated absolute accuracy, with MAD and MAPE of 0.0000, indicating that the automatic measurements were exactly aligned with the expected values. In addition, the Pearson correlation was almost perfect for examiners 1 and 2, when compared to the expected results, with an *r* value equal to 0.999. The correlation was perfect when the expected values were compared to the values automatically marked by the STEP app, with an *r value* of 1. This suggests that although the human examiners introduced small variations, the measurements consistently followed the expected pattern.

Analysis of the second part of the data, the calculations performed by STEP, based on the markings of the two examiners, and the automatic marking of the STEP system, showed small differences between the methods calculated with the manual markings and the automatic marking. For the human examiners, the MAD was 0.1111, reflecting a slight difference in relation to the expected values. The MAPE for both was very small, with a value equal to 0.0094%. However, once again, the calculations performed with the automatic marking demonstrated greater accuracy, with a MAD of 0.0100 and a MAPE of 0.0037%. In addition, the Pearson correlation followed the same pattern as the peak analysis and was perfect for the calculations performed with the automatic marking when compared to the expected theoretical values.

Table 2 shows the values of the expected results, and the findings from the marking of peaks in the frequency domain, by the examiners and by the automatic STEP system.

**Table 2:** Expected values and values obtained by the examiners using the STEP, for each marked and calculated measurement by generated frequency, in the frequency domain

Measures	Expected	Examiner 1	Examiner 2	Examiner 3*
500Hz FFT: Frequency (Hz)	500	502.07	502.07	500
500Hz FFT: Amplitude (µV)	10	10	10	10
1000Hz FFT: Frequency (Hz)	1000	998.96	998.96	1000
1000Hz FFT: Amplitude (µV)	10	10	10	10
2000Hz FFT: Frequency (Hz)	2000	1997.93	1997.93	2000
2000Hz FFT: Amplitude (µV)	10	10	10	10
FFT Sum: peak 1 Frequency (Hz)	500	502.07	502.07	500
FFT Sum: peak 1 Amplitude (µV)	10	10	10	10
FFT Sum: Peak 2 Frequency (Hz)	1000	998.96	998.96	1000
FFT Sum: Peak 2 Amplitude (µV)	10	10	10	10
FFT Sum: Peak 3 Frequency (Hz)	2000	1997.93	1997.93	2000
FFT Sum: Peak 3 Amplitude (µV)	10	10	10	10

* Examiner 3: automatic STEP marking

**Caption:** FFT - Fast Fourier Transform; Hz – hertz; µV – microvolts

**Figure 3:**
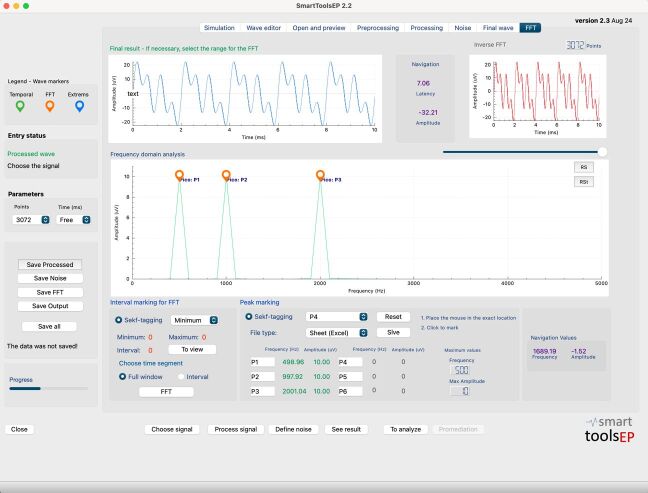
Frequency peak marking screen in the STEP application. Example of peaks identified for the composite waveform (0.5, 1, and 2 kHz)

An example of the graphs and their respective markings in the frequency domain, for the wave composed of the three sinusoids, can be found in Figure 3.

The analysis of the values found in the FFT, with the peaks marked manually and with the peaks marked automatically, when compared to the expected theoretical values, reveals minor differences between the manual and automatic measurement methods. The human examiners presented a MAD of 1.04 and a MAPE of 0.0418%, which suggests an extremely small average percentage variation in the performed measurements. The comparison of the theoretical results with the results calculated from the automatic markings of the STEP system demonstrated absolute accuracy, with a MAD and a MAPE equal to 0, evidencing that the automatic measurements were perfectly aligned with the expected values. In addition, the Pearson correlation was high for all examiners, with values equal to approximately 1 and 1, respectively, indicating a perfect correspondence between the measurements and the expected values.

An additional analysis was conducted to compare the findings of the area obtained using the integral method versus the triangle summation method. Normality analysis indicated that the data did not follow a normal distribution, justifying the use of non-parametric tests. As expected, a significant difference was observed between the areas calculated by the integral method and those calculated using the triangle method, as indicated by the Wilcoxon test, which resulted in a p-value less than 0.001. This result suggests that the two methods yield distinct values for the area under the curve. Furthermore, effect size (Cohen 's d) was calculated to assess the magnitude of the difference between the methods, yielding a value of 1.68, which reflects a large difference between the methods of calculating the areas.

### Phase II

The data were marked independently by two examiners in each of the three systems, and the mean results and confidence intervals can be found in Table 3.

**Table 3:** Latency and amplitude values of the brainstem auditory evoked potential, by click stimulus, presented by system and examiner

	AEP			Excel			STEP		
Examiner 1	**Wave I**	**Wave III**	**Wave V**	**Wave I**	**Wave III**	**Wave V**	**Wave I**	**Wave III**	**Wave V**
**Latency**									
Mean (ms)	1.45	3.55	5.33	1.43	3.57	5.3	1.42	3.53	5.29
95% CI Lower	1.41	3.47	5.19	1.39	3.48	5.18	1.38	3.45	5.16
95% CI Upper	1.49	3.63	5.47	1.47	3.66	5.42	1.46	3.61	5.42
**Amplitude**									
Mean (µV)	0.17	0.23	0.16	0.15	0.21	0.15	0.17	0.22	0.16
95% CI Lower	0.11	0.16	0.11	0.07	0.12	0.07	0.08	0.12	0.08
95% CI Upper	0.23	0.3	0.21	0.23	0.3	0.23	0.26	0.32	0.24
Examiner 2									
**Latency**									
Mean (ms)	1.45	3.57	5.31	1.44	3.55	5.31	1.41	3.47	5.27
95% CI Lower	1.41	3.49	5.17	1.4	3.45	5.19	1.37	3.37	5.14
95% CI Upper	1.49	3.65	5.45	1.48	3.65	5.43	1.45	3.57	5.4
**Amplitude**									
Mean (µV)	0.16	0.23	0.16	0.17	0.24	0.15	0.17	0.23	0.17
95% CI Lower	0.1	0.15	0.11	0.08	0.16	0.06	0.08	0.14	0.09
95% CI Upper	0.22	0.31	0.21	0.26	0.32	0.24	0.26	0.32	0.25

**Caption:** AEP - *Auditory Evoked Potential;* STEP - *Smart Tools for Evoked Potentials*; % - percentage; ms – milliseconds; µV – microvolts; CI – confidence interval

ANOVA did not reveal any significant difference between the three systems evaluated for the BAEP measures. The values found for the latencies of waves I, III and V were, respectively, F (2, 57) = 1.127 and p = 0.329, F = 1.154 and p = 0.320, and F = 0.161 and p = 0.852. For the amplitudes, the respective values were: F (2, 57) = 0.005 and p = 0.995, F = 0.013 and p = 0.987, and F = 0.129, p = 0.879.

No significant differences were found between the two examiners. The results of the t and p values for the comparison between the latencies of waves I, III and V, marked with the AEP system, were, respectively, t = -1.00 and p = 0. 334, t = -1.468 and p = 0. 164, t = 1.00 and p = 0. 334. For the Microsoft Excel system, latency values were, respectively: t = 1.00 and p = 0. 334, t = -1.00 and p = 0. 334, t = -1.468 and p = 0. 334. For the STEP, wave latency values were, respectively: t = 0. 823 and p = 0. 424, t = -0. 564 and p = 0. 582, t = -1. 871 and p = 0. 082. For amplitudes, the values for each of the three waves marked with AEP, subsequently with Microsoft Excel, and finally with STEP were, respectively, AEP: t = 1.00 and p = 0.334, t = -1.00 and p = 0.334, t = -1.00 and p = 0.334, MS Excel: -1.468 and p = 0.164, t = 0.823 and p = 0.424, t = -0.564 and p = 0.582, and STEP: t = 1.00 and p = 0.334, t = -1.00 and p = 0.334, and t = -1.00 and p = 0.334.

Table 4 presents the mean values and confidence intervals for the latencies and amplitudes of the CAEP wave markings, categorized by software and examiner.

**Table 4:** Latency and amplitude values of the cortical auditory evoked potential, elicited by speech stimuli, presented by system and examiner

	AEP			Excel			STEP		
Examiner 1	**P1**	**N1**	**P2**	**P1**	**N1**	**P2**	**P1**	**N1**	**P2**
**Latency**									
Mean (ms)	53.27	101.17	146.83	51.85	95.91	145.73	52.01	95.42	143.65
95% CI Lower	50.75	96.39	141.57	50.2	90.71	140.64	49.98	90.47	138.14
95% CI Upper	55.79	105.95	152.09	53.5	101.11	150.82	54.04	100.37	149.16
**Amplitude**									
Mean (µV)	2.19	-2.89	1.62	2.09	-2.65	1.82	2.11	-2.6	1.72
CI 95% Lower	1.41	-3.74	0.91	1.31	-3.33	1.13	1.36	-3.33	1.04
CI 95% Upper	2.97	-2.04	2.33	2.87	-1.97	2.51	2.86	-1.87	2.4
Examiner 2									
**Latency**									
Mean (ms)	53.83	99.78	148.63	52.44	96.58	147.11	52.17	98.51	146.57
95% CI Lower	49.84	94.67	143.48	48.41	91.46	141.85	48.09	93.23	141.58
95% CI Upper	57.82	104.89	153.78	56.47	101.7	152.37	56.25	103.79	151.56
**Amplitude**									
Mean (µV)	2.28	-2.97	1.66	2.19	-2.59	1.75	2.32	-2.45	1.68
CI 95% Lower	1.52	-3.77	0.96	1.42	-3.29	1.04	1.61	-3.11	0.99
CI 95% Upper	3.04	-2.17	2.36	2.96	-1.89	2.46	3.03	-1.79	2.37

**Caption:** AEP - *Auditory Evoked Potential ;* STEP - *Smart Tools for Evoked Potentials* ; % - percentage; ms – milliseconds; µV – microvolts; CI – confidence interval

Once again, the ANOVA revealed no statistically significant differences among the three systems evaluated for the CAEP wave markings. The values found for the latencies of the P1, N1 and P2 waves were, respectively, F (2, 28) = 0.528 and p = 0.594, F = 1.575 and p = 0.219, and F = 0.357 and p = 0.702. For amplitudes, the respective values were: F (2, 28) = 0.018 and p = 0.982, F = 0.165 and p = 0.849, and F = 0.080, p = 0.923.

No significant differences were found between the two examiners. The *t* and *p* values for the comparison of the latencies of waves P1, N1, and P2, marked using the AEP system, were as follows, respectively: t = -0.332 and p = 0. 745, t = 1.255 and p = 0. 230, t = -1.22 and p = 0. 242. For the Microsoft Excel system, latency values were, respectively: t = -0.320 and p = 0. 754, t = -2.09 and p = 0. 055, t = -1.692 and p = 0. 113. For the STEP system, the wave latency values were, respectively: t = -0. 093 and p = 0. 928, t = -1. 798 and p = 0. 094, t = -2. 031 and p = 0. 062. For the amplitudes the values for each of the three waves marked with AEP, followed by Microsoft Excel, and with STEP were, respectively, AEP: t = -2.064 and p = 0.058, t = 1.319 and p = 0.208, t = -1.382 and p = 0.189 , Microsoft Excel: -1.118 and p = 0.282, t = -1.581 and p = 0.136, t = 1.661 and p = 0.119, and the STEP: t = -1.520 and p = 0.151, t = -1.605 and p = 0.131, t = 0.505 and p = 0.621.

## DISCUSSION

### Phase I

The small differences observed between the expected values and the values measured by the examiners may be attributed to limitations in the sampling process. Some studies highlight that measurement accuracy can be significantly influenced by the sampling rate used. The equivalent sampling technique can be an efficient solution to improve accuracy without requiring extremely high sampling rates, which is corroborated by studies that discuss the effectiveness of this approach in situations with high harmonic interference^(8,9)^.

Although higher sampling rates can potentially reduce errors, they increase the complexity and processing requirements of the system, which may not be feasible for all equipment and applications, especially in systems with processing limitations^(10-12)^. Therefore, the sampling rates currently used in standard systems are considered sufficient to ensure accurate measurements in most applications; and increasing these rates should be carefully considered to avoid processing overhead and stability issues.

The minimal differences found between the expected results and those obtained by the STEP software (both in manual and automatic measurements) validate the use of this system for the analysis of electrophysiological signals. Previous studies have shown that minor variations in measurements are common and may result from inherent limitations in the sampling process and the specific characteristics of the equipment used^(8)^. Even with advanced technologies such as calcium imaging and electrophysiology, measurement differences may emerge due to variability in data acquisition and processing methods^(13)^.

Furthermore, the routine clinical practice of comparing electrophysiological exam results with standards acquired on the same equipment and analysis system mitigates the impacts of these variations. Different equipment, even when connected to the same person, simultaneously, with the same protocol and in the same setting, can yield different electrophysiological tracings due to differences in calibration and signal processing algorithms^(8)^. Therefore, the small variations observed in the STEP measurements, even when slightly larger than the expected results, would likely affect both the standard and the analyzed exam, therefore allowing for valid comparisons.

The use of the sum of two triangles^^2^^, or a single triangle^^3^^, or a rectangle^^4^ (14)^, as well as the displacement of the wave V’s peak to the baseline and the calculation of a rectangle formed by the product of amplitude values and the time interval between sampling points, from the peak of wave V to the trough of wave A^(15)^ can be found for calculating the area under the curve between the peak of wave V and the trough of wave A in electrophysiological assessments such as frequency-following response (FFR) and other measurements.

However, even the most refined method, the sum of two triangles, is not ideal for accurate sinusoidal waveforms, and even less suitable for analyzing irregular electrophysiological data. According to the literature, integration methods, such as the use of definite integrals, are preferable for calculating the area with greater precision, as they more accurately capture waveform variability^(16)^. The use of triangle or rectangle approximations may lead to underestimation or overestimation of the actual area, especially in curves with significant fluctuations. This is corroborated by studies showing that methods relying on simple geometric approximations often present errors^(17)^, reinforcing that the two-triangle method is not the best option. Effective methods may use combinations of geometric forms to calculate the area under the curve (AUC), within the range defined by Cartesian coordinates, calculating the area from the maximum value to zero and from the minimum value to zero, in modules and summed, to increase measurement accuracy^(17)^.

### Phase II

The results of the study with patient examinations indicated that there were no significant differences in latencies and amplitudes of the BAEP and CAEP waves marked by two examiners using three different systems, a clinical gold standard (AEP), another gold standard used in mathematical analyses (Microsoft Excel), and the new proposed application (STEP). No significant differences were found in the aforementioned markings between the examiners.

In the literature, several studies report the existence of small variations in amplitudes and latencies between the different equipment and systems used for the assessment of auditory evoked potentials^(8)^, and due to the diverse protocols used^(18)^. In this phase, the present study used exams performed on the same equipment and with the same protocol, whose waveforms were marked in three different systems. Thus, the fact that no differences were found among such markings indicates that the STEP application can be used clinically or in research, as it has similar results to the two tested gold standard systems.

Thus, if differences had been observed between examiners, these would merely indicate a lack of standardization in the technique used for marking the recordings. Consistency between different examiners when measuring latencies and amplitudes of BAEP and CAEP waves is crucial for data reliability^(19)^. The present study found no significant differences between examiners, which is in agreement with the literature, which points to the reliability of auditory evoked potential measurements when performed by different professionals trained with the same technique. This is a positive indicator, as it reveals that adequate training of examiners can minimize measurement variability^(20)^, ensuring consistency of results regardless of the professional performing the markings.

Finally, two limitations of the study and the actions taken to minimize them can be highlighted: 1. The use of simulated signals may not capture the full complexity of actual electrophysiological signals. To mitigate this limitation, the parameters of the generated waves were carefully defined to reflect ideal measurement conditions, and the accuracy was compared with rigorous theoretical calculations. In addition, actual data were subsequently analyzed. 2. When validating automatic and manual measurements, the risk of discrepancies due to human error or limitations of the application algorithms is present. To minimize this limitation, the study included reviews by highly qualified mathematicians and computer professionals, the comparison of results between two experienced examiners, as well as the use of a third automatic measurement, which increases data reliability.

## CONCLUSION

The STEP demonstrated high accuracy in marking peaks and troughs, as well as in calculating the slope and the area under the curve between two points, both through definite integration and through approximation using the area of two triangles, when comparing theoretical and experimental results. In the analysis of real exams, no significant differences were found in the latencies and amplitudes of BAEP and CAEP waves marked using the STEP application, when compared with widely used gold-standard applications available on the market.

## Footnotes

1 All calculations were reviewed by a mathematician.

2

$\begin{array}{l} \left[\frac{\left(\left|amplitude_{V}\right|\right)*\left(zero-cross_{latency}-V_{latency}\right)}{2}\right]+\\ \left[\frac{\left(\left|amplitude_{A}\right|\right)*\left(A_{latency}-zero-cross_{latency}\right)}{2}\right] \end{array}$

3

$\frac{\left(\left|amplitude_{V}\right|+\left|amplitude_{A}\right|\right)*\left(Latency_{A}-Latency_{V}\right)}{2}$

4

$\left(\left|amplitude_{V}\right|+\left|amplitude_{A}\right|\right)*\left(Latency_{A}-Latency_{V}\right)$

## Appendix I

Software availability

Information about the modeling package software is as follows:

1. Software name: STEP wave generator.

2. Developer contact: Pedro de Lemos Menezes pedro.menezes@uncisal.edu.br

3. Address: Rua Doutor Jorge de Lima, 113. Trapiche da Barra - Zip Code: 57010-300, Maceió, AL - Brazil.

4. Phone number: +55 (82) 3315-6701

5. Year of first availability: 2024.

6. Suggested hardware: 2.6GHz *Intel Core i7 processor, with 16GB 1600MHz DDR3 and 1TB HDD. However, we ran the simulations on an* Apple M1 computer, 8-core CPU, 8-core GPU, 16-core *Neural Engine. M*acOS *Sonoma* 14.0 operating system.

7. *Software*: The entire project is running on Ubuntu 16.04 (recommended)

8. Availability: Available on Github3: https://github.com/pedrolemosmenezes/Smart-Tools-Evoked-Potentials/tree/ac1b231a46d3b0fa90491d58a5d5bc1bd3ec756d

9. Cost: All tools free.
