# ERRATA

**DOI:** 10.1590/2317-1782/e20250133pt

**Published:** 2025-09-24

Por conta de uma falha no processo de produção editorial, o artigo “Validação de aplicativo de distribuição gratuita para análise de sinais eletrofisiológicos: Smart Tools for Evoked Potentials (STEP)” (DOI https://doi.org/10.1590/2317-1782/e20240265pt), publicado em CoDAS, 37(4), e20240265, 2025, com um erro em uma equação.

Na página 4, onde se lia:


$$Slope:Slope_{1kHz}=\frac{20}{0.5}=40V/ms$$

Leia-se:


$$Slope:Slope_{1kHz}=\frac{20}{0.5}=40\mu V/ms$$

