# Supplementary material for: Validation of a freely distributable software for the analysis of electrophysiological signals: Smart Tools for Evoked Potentials (STEP)
Source: Codas. 2025 Aug 8;37(4):e20240265. doi: 10.1590/2317-1782/e20240265en (PMC12337715; doi:10.1590/2317-1782/e20240265en)
Supplement: Translation

# Translation: Validação de aplicativo de distribuição gratuita para análise de sinais eletrofisiológicos: Smart Tools for Evoked Potentials (STEP)

**DOI:** 10.1590/2317-1782/e20240265pt

**Authors:** Kelly Cristina Lira de Andrade, Aline Tenório Lins Carnaúba, Carlos Henrique Alves Batista, Danielle Cavalcante Ferreira, Raí Fernandes Santos, Raquel da Silva Cabral, Pedro de Lemos Menezes

**Affiliations:** 1 Programa Associado de Pós-graduação em Fonoaudiologia – PPGFON (UFPB/UFRN/UNCISAL), Universidade Estadual de Ciências da Saúde de Alagoas – UNCISAL - Maceió (AL), Brasil.; 2 Laboratório de Computação Científica e Análise Numérica, Universidade Federal de Alagoas – UFAL - Maceió (AL), Brasil.

**Keywords:** Audiologia, Potenciais Evocados, Potenciais Evocados Auditivos, Estudo de Validação, Processamento de Sinais Assistido por Computador

## Abstract

**Objetivo:**

O presente estudo teve como objetivo validar a ferramenta STEP, um aplicativo desenvolvido para a análise dos diversos sinais eletrofisiológicos auditivos e vestibulares. O STEP foi projetado para fornecer maior precisão e eficiência na análise de latências, amplitudes, e outras características morfológicas das ondas, como cálculo da área, slope e transformada rápida de Fourier.

**Método:**

A metodologia foi dividida em duas etapas, uma comparou traçados simulados e outra dados experimentais. Na primeira etapa, foram geradas ondas por meio de funções matemáticas e suas características foram marcadas e analisadas por examinadores humanos e pelo próprio aplicativo. Na segunda etapa, o STEP foi testado em exames eletrofisiológicos reais, comparando-se as latências e amplitudes das ondas entre o aplicativo em questão e outros dois sistemas padrão ouro.

**Resultados:**

Os resultados demonstraram alta precisão do STEP, tanto na marcação manual quanto automática dos picos e vales, e nos cálculos subsequentes. Foi observado, ainda, que não há diferenças significativas entre os sistemas utilizados. Também não foram encontradas diferenças entre os examinadores.

**Conclusão:**

o STEP é uma ferramenta precisa para a realização da marcação das latências e amplitudes das ondas dos potenciais eletrofisiológicos, bem como para a realização dos cálculos da área P1N1, do slope e da transformada rápida de Fourier.

## INTRODUÇÃO

Nos últimos anos, o avanço das tecnologias da informação tem permitido o desenvolvimento de novas ferramentas que visam aprimorar a eficiência e a precisão das análises clínicas. Diversos estudos têm destacado a importância de validar estas novas tecnologias antes de sua implementação para realização de pesquisas, e na prática clínica^(1,2)^. Um estudo, por exemplo, observou o uso da inteligência artificial, por meio de um algoritmo de análise de sinais cardíacos, demonstrando a importância de tais comparações para garantir a confiança nos novos métodos^(3)^. Outro estudo, por outro lado, validou um software de análise de eletroencefalograma (EEG), comparando seus resultados com os de um sistema padrão-ouro amplamente utilizado^(4)^. Além disso, a validação de um software de análise de respostas eletrofisiológicas auditivas, evidenciou a necessidade de ferramentas precisas para a prática clínica na Audiologia^(5)^. Apesar da existência de alguns estudos, ainda são poucas as iniciativas para análise mais aprofundada das ondas eletrofisiológicas auditivas que transcendam a simples marcação das latências, amplitudes e intervalos interpicos.

Nesse contexto, podemos destacar os exames eletrofisiológicos, mais especificamente o PEATE (Potenciais Evocados Auditivos de Tronco Encefálico), onde a precisão na marcação das latências e amplitudes das ondas é crucial para análise correta dos resultados e os seus devidos encaminhamentos. No entanto, as medições convencionais de latência e amplitude podem não capturar toda a complexidade das ondas eletrofisiológicas. Estudos têm apontado a relevância de análises morfológicas mais detalhadas, como o cálculo da área sob a curva, o slope e a transformada rápida de Fourier, que permitem uma compreensão mais profunda do funcionamento neurológico e auditivo, o que aumenta a precisão nos laudos e conclusões dos exames^(6,7)^.

Na literatura, é possível encontrar, ainda, um trabalho que observou pequenas diferenças de latência e amplitude em exames eletrofisiológicos realizados em equipamentos de marcas diferentes^(8)^. No material complementar de um dos estudos, por exemplo, os pesquisadores gravaram simultaneamente um único exame com três sistemas distintos (IHS, *Bio-logic Navigator Pro* e *Compumedics Neuroscan System*). Apesar de utilizarem os mesmos pacientes, mesmo protocolo, montagem de eletrodos e condições ambientais, foram observadas variações nas latências e amplitudes do frequency following response (FFR), evidenciadas, principalmente, nos componentes A, C e D^(8)^. Esses resultados indicam que, embora as morfologias das ondas sejam aparentemente semelhantes, as características técnicas específicas de cada equipamento, como seus algoritmos e hardwares, podem influenciar nos resultados.

Diante desse cenário, esse trabalho propõe a validação da ferramenta *Smart Tools for Evoked Potentials* (STEP), um software desenvolvido por uma Universidade pública do Brasil e projetado para aumentar os recursos de análises morfológicas de todos os exames eletrofisiológicos auditivos e vestibulares frequentemente utilizados na avaliação auditiva. O aplicativo fornece uma maior precisão e eficiência na análise de latências e amplitudes das ondas, e na realização de outras análises morfológicas, a exemplo do cálculo da área, do *slope* e da transformada rápida de Fourier. Espera-se fornecer evidências sólidas que suportem a adoção do aplicativo como uma ferramenta válida e eficaz para profissionais de saúde, e da Audiologia em especial.

## MÉTODO

### Smart Tools Evoked Potentials (STEP)

O aplicativo possui três ferramentas principais que propiciam, respectivamente, o ensino da eletrofisiologia e o treinamento de profissionais; a marcação e a análise das respostas de qualquer potencial eletrofisiológico realizado na avaliação auditiva; e a realização de pesquisas.

A primeira ferramenta é constituída por um sistema capaz de simular ondas eletrofisiológicas, estudar as repercussões das mudanças de cada parâmetro do protocolo nos traçados ondas, tais como, filtros, taxa de estimulação, tipos de estímulos etc.

A segunda função, objeto do presente estudo, é capaz de fazer upload dos exames eletrofisiológicos de qualquer paciente, exibir os exames num gráfico, permitir a marcação dos picos e vales das ondas eletrofisiológicas, fazer a auto marcação desses picos e vales, calcular latências, amplitudes, intervalos interlatências e interamplitudes, calcular o slope e a área entre dois pontos utilizando várias técnicas diferentes, cálculo de ângulos, razão das áreas, cálculo do *Mismatch Negativity* (MMN), cálculo de índices de assimetria e cálculo do componente de interação binaural (BIC). Além disso, nessa função ainda é possível ser realizada a transformada rápida de Fourier de toda a janela, de um intervalo de tempo e a marcação dos picos de amplitude para cada frequência, de forma manual e automática.

A terceira função, serve para a realização de pesquisas, facilita a tabulação dos dados em planilha Excel, testa protocolos de pesquisa e oferece suporte para realização de análises estatísticas.

O aplicativo foi desenvolvido na linguagem de programação C++, com o auxílio da ferramenta QT 6.3, para macOS 14.5. Posteriormente foi desenvolvida uma versão para Windows. Atualmente o STEP encontra-se na versão 2.6 para os dois sistemas operacionais.

### Metodologia do estudo

O presente estudo refere-se à validação de parte das medidas realizadas pelo aplicativo STEP, estabelecendo-se a precisão do mesmo para realizar tal processamento. Além disso, uma das motivações para o desenvolvimento do STEP foi a democratização do acesso a tecnologias avançadas de análise clínica na área. Por isso, o aplicativo será disponibilizado gratuitamente para outras instituições públicas, incluindo universidades, hospitais, institutos de pesquisa, e outros. A metodologia do presente estudo priorizou uma abordagem metodológica rigorosa, incluindo comparações teóricas, experimentais e práticas. Foram realizadas validações das medidas no domínio do tempo e do domínio da frequência. No primeiro caso, foram validadas: (i) a marcação das latências e amplitudes, manuais e automáticas; (ii) o cálculo das áreas abaixo da curva entre dois pontos, utilizando a técnica da integral e da soma de dois triângulos; (iii) e o cálculo do slope. No segundo caso, foram validadas: (i) as marcações dos picos de frequências e (ii) seus respectivos valores de amplitude, de forma manual e automática.

A metodologia do estudo está organizada em duas etapas principais, ilustrada na Figura 1, e detalhada a seguir.

**Figura 1:** Resumo esquemático da metodologia

**Etapa I.** Comparar os resultados obtidos, utilizando-se o aplicativo STEP, com os resultados esperados, por meio de cálculos matemáticos^^1^^.

**1. Geração das ondas:** Inicialmente foram geradas no *Phyton* versão 3.10.1 para macOS 14.5 (Apêndice I), e salvas no formato MS Excel, três ondas a partir de funções seno e uma quarta que representava o somatório das três. As ondas senoidais no domínio do tempo foram geradas usando-se a equação:


$$y\left(t\right)\;=\;A\;sin\left(2\;\pi \;f\;t\right)$$ (1)

Onde:

$y\left(t\right)$ representa o valor da onda gerada em um instante t,

A é a amplitude que representa a altura máxima que a onda atinge com uma linha de base referencial,

sin é a função trigonométrica seno que descreve a oscilação periódica da onda,

$2\pi \;\left(rad\right)$ representa uma volta completa no ciclo trigonométrico,

f é a frequência da onda, i.e., o número de ciclos completos que a onda realiza em 1 segundo.

Os parâmetros utilizados foram: frequências de 0.5 kHz, 1 kHz e 2 kHz; intervalo de tempo de 0 ms a 10 ms; amplitude do pico: 10 μV; amplitude do vale: -10 μV; amplitude pico a vale: $10-\left(-10\right)=20\;\mu V$; foram geradas 3072 coordenadas cartesianas $\left(x,\;y\right)$, para cada uma das ondas. Dessa forma, foram armazenados o tempo $\left(t\right)$, variando de 0 a 10 ms; amplitudes para $0.5\;kHz\;\left(y1\right)$; amplitudes para $1\;kHz\;\left(y2\right)$; amplitudes para $2\;kHz\;\left(y3\right)$; somatório das três ondas $\left(y4\;=\;y1+y2+y3\right)$.

## CÁLCULO DOS VALORES ESPERADOS NO DOMÍNIO DO TEMPO

### Funções matemáticas utilizadas

#### Identificação matemática dos Picos e Vales

- Para uma onda senoidal y(*t*) = *A sin(2 π f t)*, os primeiros picos e vales ocorrem nas seguintes condições: pico: $t_{pico\;1}=\;\frac{1}{4f}$ ; vale: $t_{vale\;1}=\;\frac{3}{4f}\;$; cruza-zero: $t_{cruza-zero}=\;\frac{1}{2f}\;$;

#### Cálculo da área P1N1 absoluta abaixo da curva

A área abaixo da curva representa a soma total dos valores sob uma curva em um gráfico. Para calcular essa área, pode-se utilizar equações integrais. Essa medida é útil para entender a magnitude de fenômenos, como a quantidade total de um sinal ao longo do tempo.

A área $A_{area}$ abaixo da curva entre o primeiro pico e o primeiro vale pode ser calculada pela integral da função seno entre o $t_{pico\;}$e o $t_{vale\;}$:


$$A_{area}=\;\overset{t_{vale\;}}{\underset{t_{pico\;}}{\int }}A\;sin\left(2\pi ft\right)\;dt$$ (2)

Utilizando as identidades trigonométricas e a integral da função seno:


$$\;A_{area}=-\frac{A}{2\pi f}cos(2\pi ft)\{t_{vale\;}\;t_{pico\;}\;$$ (3)

Substituindo os valores $t_{pico\;}$e $t_{vale\;}$:


$$\;A_{area}=-\frac{A}{2\pi f}[cos(2\pi ft_{vale\;})-\left(2\pi ft_{pico\;}\right)]$$ (4)

#### Cálculo da área P1N1 abaixo da curva, aproximada por dois triângulos

A área também pode ser calculada aproximadamente pela soma da área de dois triângulos.


$$A_{area\;dois\;triângulos}=\;A_{area\;triângulo\;1}+\;A_{area\;triângulo\;2}$$ (5)

$$A_{area\;triângulo\;1}=\;\frac{\begin{array}{l} \left(intervalo\;de\;tempo\;entre\;pico\;e\;zero(instante\;de\;cruzamento\right))\\ \times \;\left(amplitude\;do\;pico\right) \end{array}}{2}$$ (6)

$$A_{area\;triângulo\;2}=\;\frac{\begin{array}{l} \left(intervalo\;de\;tempo\;entre\;zero\left(instante\;de\;cruzamento\right)\;e\;o\;vale\right)\\ \times \;\left(amplitude\;do\;vale\right) \end{array}}{2}$$ (7)

#### Cálculo do Slope

O slope (ou inclinação) é a medida de quão rapidamente uma variável muda em relação a outra. Em uma linha reta em um gráfico, o slope é calculado como a diferença na altura (y) dividida pela diferença na base (t). A fórmula é:


$$Slope=\frac{\Delta y}{\Delta t}=\frac{variação\;da\;amplitude\;entre\;o\;pico\;e\;o\;vale}{Intervalo\;de\;tempo\;entre\;o\;pico\;e\;o\;vale}$$ (8)

### Valores encontrados para cada uma das frequências

Cálculos dos valores t e y(t) para f igual a 0.5 kHz, 1 kHz e 2 kHz, considerando a amplitude de -10 a 10 microvolts, para pico, vale e cruzamento em zero:

#### Frequência de 0.5 kHz


$$pico:t=\frac{1}{4f}=\frac{1}{4X500}=\frac{1}{2000}=0.0005\;s$$ (9)

$$pico:t_{pico\;1}=0.5\;ms;$$ (10)

$$\begin{array}{l} y\left(t\right)=Asin\left(2\pi ft\right)=y\left(0.0005\right)=10sin(2\pi 500x0.0005)=\\ 10sin(2\pi 0.25)=10sin(\frac{\pi }{2})=10\;\mu V \end{array}$$ (11)

$$vale:t=\frac{3}{4f}=\frac{3}{4X500}=\frac{3}{2000}=0.0015\;s$$ (12)

$$vale:t_{vale\;1}=\;1.5\;ms$$; (13)

$$\begin{array}{l} y\left(t\right)=Asin\left(2\pi ft\right)=y\left(0.0015\right)=10sin(2\pi 500x0.0015)=\\ 10sin(2\pi 0.75)=10sin(\frac{3\pi }{2})=-10\;\mu V \end{array}$$ (14)

$$Cruzamento\;em\;zero:t=\frac{1}{2f}=\frac{1}{2X500}=\frac{1}{1000}=0.001\;s$$ (15)

$$cruza-zero:t_{P1N1}=1\;ms.$$ (16)

$$\begin{array}{l} y\left(t\right)=A\sin\left(2\pi ft\right)=y\left(0.001\right)=10\sin(2\pi 500X0.001)=\\ 10\sin(2\pi 0.5)=10\sin(\pi )=\;0\;\mu V \end{array}$$ (17)

Área absoluta abaixo da curva*:*$A_{area\;1}=\;\overset{1.5\;ms}{\underset{0.5\;ms}{\int }}10\;sin\left(2\pi 500t\right)\;dt=6.36$ μVms*.* Área P1N1 abaixo da curva, aproximada por dois triângulos:


$$A_{area\;triângulo\;1}=\;\frac{\left(1-0.5\right)\times \;\left(10\right)}{2}=2.5\;\mu Vms$$ (18)

$$A_{area\;triângulo\;2}=\;\frac{\left(1.5-1\right)\times \;\left(10\right)}{2}=2.5\;\mu Vms$$ (19)

$$A_{area\;dois\;triângulos}=5\;\mu Vms$$ (20)

$$Slope:Slope_{0.5kHz}=\frac{20}{1}=20\mu V/ms$$ (21)

#### Frequência de 1 kHz

Picos: pico: $t_{pico\;1}=0.25\;ms;$ vale: $t_{vale\;1}=\;0.75\;ms$*;* cruza-zero: $t_{P1N1}=0.50\;ms;$ Área absoluta abaixo da curva: $A_{area\;1}=\;\overset{0.75\;ms}{\underset{0.25\;ms}{\int }}10\;sin\left(2\pi 1000t\right)\;dt=3.18$ μVms*;* Área P1N1 abaixo da curva, aproximada por dois triângulos:


$$A_{area\;triângulo\;1}=\;\frac{\left(0.50-0.25\right)\times \;\left(10\right)}{2}=1.25\;\mu Vms$$ (22)

$$A_{area\;triângulo\;2}=\;\frac{\left(0.75-0.50\right)\times \;\left(10\right)}{2}=1.25\;\mu Vms$$ (23)

$$A_{area\;dois\;triângulos}=2.5\;\mu Vms$$ (24)

$$Slope:Slope_{1kHz}=\frac{20}{0.5}=40\mu V/ms$$ (25)

#### Frequência de 2 kHz

Picos: pico: $t_{pico\;1}=0.125\;ms;$ vale: $t_{vale\;1}=\;0.375\;ms$*;* cruza-zero: $t_{P1N1}=0.25\;ms;$ Área absoluta abaixo da curva: $A_{area\;1}=\;\overset{0.375\;ms}{\underset{0.125\;ms}{\int }}10\;sin\left(2\pi 2000t\right)\;dt=1.59$ μVms*;* Área P1N1 abaixo da curva, aproximada por dois triângulos:


$$A_{area\;triângulo\;1}=\;\frac{\left(0.25-0.125\right)\times \;\left(10\right)}{2}=0.625\;\mu Vms$$ (26)

$$A_{area\;triângulo\;2}=\;\frac{\left(0.375-0.25\right)\times \;\left(10\right)}{2}=0.625\;\mu Vms$$ (27)

$$A_{area\;dois\;triângulos}=1.25\;\mu Vms$$ (28)

$$Slope:Slope_{2kHz}=\frac{20}{0.25}=80\mu V/ms$$ (29)

## CÁLCULO DOS VALORES ESPERADOS NO DOMÍNIO DAS FREQUÊNCIAS

A análise no domínio das frequências envolve a transformação de sinais do domínio do tempo para o domínio das frequências, permitindo identificar as componentes de frequência presentes em um sinal composto. Para realizar essa análise, foi utilizada a transformada de Fourier, que decompõe uma função de tempo em suas componentes senoidais de diferentes frequências.

### Transformada de Fourier

A Transformada de Fourier é uma ferramenta matemática que transforma um sinal contínuo no tempo em um sinal contínuo no domínio das frequências. A fórmula da transformada de Fourier para uma função $y\left(t\right)$ é dada por:


$$Y\left(f\right)=\overset{\infty }{\underset{-\infty }{\int }}y\left(t\right)e^{-j2\pi ft}dt$$ (30)

onde:

$Y\left(f\right)$ é a Transformada de Fourier de $y\left(t\right)$,

j é a unidade imaginária $\left(\sqrt{-1}\right)$

f é a frequência,

t é o tempo.

Para sinais discretos, como no caso dos dados gerados para este experimento, a transformada de Fourier discreta (DFT) é utilizada, sendo computada através do algoritmo da transformada rápida de Fourier (FFT).

#### Aplicação da Transformada de Fourier

Para analisar as frequências presentes no sinal somatório $y_{4}\left(t\right)$ (resultado da soma das três ondas senoidais), aplicamos a FFT aos dados do sinal. A FFT transforma o sinal do domínio do tempo para o domínio das frequências, revelando as componentes de frequência e suas amplitudes. Considerando que o sinal $y_{4}\left(t\right)$ é uma soma de três ondas senoidais com frequências de 0.5 kHz, 1 kHz e 2 kHz, e que cada uma dessas ondas possui uma amplitude de pico a pico de 20 μV, é esperado picos de amplitude no espectro de frequências correspondentes a estas frequências.

### Valores encontrados no domínio das frequências

Após a aplicação da FFT, os resultados no domínio das frequências são representados em um gráfico onde o eixo horizontal (x) representa a frequência e o eixo vertical (y) representa a amplitude correspondente.

Assim, é esperado encontrar, após a aplicação da FFT, um pico em cada uma das ondas separadas (0.5 kHz, 1 kHz e 2 kHz), exatamente nas mesmas frequências, e os três picos juntos na onda da soma (y_4_). Todos os picos esperados, padronizados, devem ter 10 μV de amplitude, como será visto a seguir. Esses picos foram marcados por dois examinadores experientes, e também de forma automática, utilizando-se uma das funções disponíveis no STEP. Para uma onda senoidal $y\left(t\right)=A\;sin\left(2\pi ft\right)$, onde *A* é a amplitude de pico e *f* é a frequência. A amplitude observada no espectro de frequência após a FFT é:


$$A_{freq}=\;\frac{A_{pico\;a\;pico}}{2}$$ (31)

onde:


$$A_{pico\;a\;pico}=20\;\mu V\;\left(10\;\mu V\;a-10\;\mu V\right)$$ (32)

Assim, a amplitude esperada no domínio das frequências para a componente de 1 kHz é:


$$A_{freq}=\;\frac{20\;}{2}=10\;\mu V$$ (33)

Portanto, após aplicar a FFT, espera-se que a amplitude da componente de cada uma das frequências seja de 10 μV.

## COMPARAÇÃO DOS RESULTADOS ESPERADOS MATEMATICAMENTE COM OS RESULTADOS OBTIDOS EXPERIMENTALMENTE NO APLICATIVO STEP

Dois examinadores treinados marcaram manualmente os picos e vales das ondas geradas, utilizando o aplicativo STEP. Além das medidas dos examinadores, também foi realizada uma terceira medida, utilizando-se a função de marcação automática do referido aplicativo. Esta medida automática foi considerada o terceiro examinador. Após a marcação dos picos, utilizada a “função calcular” do aplicativo, a qual conduz automaticamente os cálculos das latências, amplitudes, intervalos interpicos, áreas, slopes entre outros. Esse processo foi repetido para cada uma das três senoides analisadas. Posteriormente foi utilizada a função FFT do aplicativo, para cada uma das três frequências e para a soma delas.

Para comparar os dados teóricos com os dados marcados pelos examinadores e automaticamente pelo STEP (terceiro examinador), foram utilizadas as seguintes métricas:

A diferença média absoluta (MAD), métrica utilizada para calcular a média das diferenças absolutas entre os picos e vales teóricos e os valores obtidos com o aplicativo. Essa técnica é útil para avaliar a precisão dos valores estimados. Como se baseia em diferenças absolutas, a MAD é robusta contra grandes desvios e oferece uma visão clara do quanto, em média, os valores observados diferem dos teóricos, independentemente de outliers;
O erro percentual médio absoluto (MAPE), por sua vez, foi utilizado para medir a precisão relativa das medidas ao calcular a média das diferenças percentuais absolutas entre os valores estimados e os teóricos. Essa técnica é amplamente utilizada por expressar o erro em termos percentuais, facilitando a interpretação e a comparação entre diferentes conjuntos de dados. O MAPE oferece uma visão direta de quão próximas, em termos percentuais, as medições estão dos valores esperados, sendo especialmente útil para avaliar a eficácia de modelos ou ferramentas de medição;
O coeficiente de correlação de Pearson foi utilizado para avaliar a correlação entre os valores teóricos e os valores obtidos com o aplicativo;
O coeficiente de correlação intraclasse (ICC) para avaliar a concordância intraexaminador e interexaminador.

**Etapa II.** Comparação dos traçados eletrofisiológicos, potenciais evocados auditivos de tronco encefálico (PEATE) e potenciais evocados auditivos corticais (PEAC), marcados por dois examinadores experientes no sistema do padrão ouro clínico (AEP), no sistema padrão ouro para cálculos matemáticos (Microsoft Excel) e no STEP.

Para a comprovação da eficácia nas medidas realizadas pelo STEP, seria necessário apenas comprovar sua precisão com ondas matematicamente controladas, como realizado na etapa I. Contudo, para uma avaliação efetiva de dados eletrofisiológicos reais, e como comprovação adicional, optou-se por avaliar o aplicativo na análise do PEATE, por ser o potencial de curta latência mais amplamente utilizado nas avaliações eletrofisiológicas auditivas, e o PEAC, por ser um dos potenciais de longa latência mais utilizados na área. O objetivo desta etapa foi comparar registros dos potenciais eletrofisiológicos realizados no aplicativo AEP (versão 7.3) do sistema Biologic Pro Navigator, os quais foram posteriormente convertidos em arquivos TXT, na forma de dados separados por vírgula (CSV), com o auxílio do aplicativo ASCII (versão 2.0) disponibilizado pela Biologic. O sistema utilizado foi considerado o padrão ouro e é usualmente utilizado na prática clínica da área em todo o mundo. Os traçados foram marcados por dois examinadores clínicos com larga experiência na marcação de exames eletrofisiológicos, no próprio aplicativo onde os dados foram adquiridos (AEP *Biologic*), no *MS Excel* e no aplicativo em análise (STEP). Posteriormente os dados foram comparados por meio de técnicas estatísticas.

Inicialmente, 15 exames foram selecionados por amostragem aleatória simples entre os exames eletrofisiológicos do banco de dados de uma Universidade pública. Foram extraídas as informações do registro do traçado, além de outras informações secundárias: o sexo e a idade do paciente e condição do paciente, obrigatoriamente sem alteração auditiva, apenas para confirmar os critérios de inclusão dos exames a serem avaliados. Esses traçados foram exportados para o formato TXT usando a plataforma do equipamento clínico utilizado. Posteriormente, os arquivos foram importados para o *MS Excel* e para o STEP. A marcação foi realizada em momentos distintos e aleatórios. Assim, dois examinadores clínicos experientes marcaram manualmente os picos e vales das ondas nos três aplicativos, em ordem aleatória, registrando as latências e amplitudes das mesmas. Finalmente foram calculadas a média, desvio padrão e os intervalos de confiança de 95% das latências e amplitudes, marcadas por ambos os examinadores em cada aplicativo. Foi utilizado o teste o ANOVA de medidas repetidas para cada variável, no PEATE (latências e amplitudes das ondas: I, III e V) e no PEAC (latências e amplitudes das ondas: P1, N1 e P2), para verificar se existiam diferenças significativas entre as ondas marcadas os diferentes sistemas (AEP, *MS Excel*, STEP). Além disso, foi aplicado o teste T de *Student* para medidas emparelhadas, com correção de *Bonferroni*, para observar se existiam diferenças significativas entre os examinadores 1 e 2. O valor t foi utilizado para observar a magnitude da diferença observada entre eles.

Finalmente as medidas das áreas utilizando os dois métodos testados, integral abaixo da curva e soma dos dois triângulos foram comparadas utilizando-se o teste de Wilcoxon, e o tamanho dos efeitos calculados pelo teste d de *Cohen*.

A comparação entre os três sistemas avaliados foi realizada por meio do teste ANOVA, com o valor F representando a razão entre a variação entre grupos e a variação dentro dos grupos.

Todos os testes estatísticos foram conduzidos com o auxílio do aplicativo IBM SPSS versão 28, para macOS versão *Sonoma* 14.5. A normalidade das amostras foi calculada por meio do teste de *Shapiro-wilk* e a esfericidade por meio do teste de *Mauchly*. Para fatores não esféricos, o valor de p foi obtido com o teste de *Greenhouse-Geisser*. Os valores foram considerados significativos quando p < 0.05. O valor de beta estabelecido foi de 0.1.

## RESULTADOS

### Etapa I

Os resultados esperados, matematicamente, para as latências e amplitudes dos picos e vales das ondas, cruza-zerotime, áreas P1N1 abaixo da curva, área da soma dos dois triângulos e slope encontrados, por frequência, podem ser observados na Tabela 1, a seguir. Na referida tabela, ainda estão dispostos os valores dessas medidas encontrados experimentalmente, com a ajuda do STEP, para cada um dos examinadores e para a marcação automática do STEP (examinador 3).

**Tabela 1:** Valores esperados e encontrados pelos examinadores utilizando o STEP, para cada medida, por frequência gerada, no domínio do tempo

Medidas	Esperado	Examinador 1	Examinador 2	Examinador 3*
500 Hz pico 1: Latência (ms)	0,5	0,5	0,5	0,5
1000 Hz pico 1: Latência (ms)	0,25	0,25	0,25	0,25
2000 Hz pico 1: Latência (ms)	0,12	0,12	0,12	0,12
Todas as frequências pico 1: Amplitude (µV)	10	10	10	10
500 Hz vale 1: Latência (ms)	1,5	1,5	1,5	1,5
1000 Hz vale 1: Latência (ms)	0,75	0,74	0,74	0,75
2000 Hz vale 1: Latência (ms)	0,37	0,37	0,37	0,37
Todas as frequências vale 1: Amplitude (µV)	-10	-10	-10	-10
500 Hz: cruza-zero(ms)	1	1	1	1
1000 Hz: cruza-zero(ms)	0,5	0,5	0,5	0,5
2000 Hz: cruza-zero(ms)	0,25	0,25	0,25	0,25
500 Hz: Área abaixo da curva(µV*ms)	6,36	6,33	6,33	6,33
1000 Hz: Área abaixo da curva(µV*ms)	3,18	3,05	3,05	3,15
2000 Hz: Área abaixo da curva(µV*ms)	1,59	1,56	1,56	1,56
500 Hz: Área de dois triângulos(µV*ms)	5	5	5	5
1000 Hz: Área de dois triângulos(µV*ms)	2,5	2,5	2,5	2,5
2000 Hz: Área de dois triângulos(µV*ms)	1,25	1,25	1,25	1,25
500 Hz: Slope (µV/ms)	20	20	20	20
1000 Hz: Slope (µV/ms)	40	40,81	40,81	40
2000 Hz: Slope (µV/ms)	80	80	80	80

* Examinador 3: marcação automática do STEP

**Legenda:** Hz – hertz; ms – milissegundos; µV – microvolts

A seguir, na Figura 2, pode ser encontrado um exemplo das ondas marcadas com o auxílio do aplicativo STEP, para a frequência de 0.5 kHz.

**Figura 2:** Tela de marcação de picos e vales do aplicativo STEP. Exemplo de traçado marcado para a onda de 0.5 kHz

Análise apenas das marcações das ondas, picos e vales, e suas respectivas latências e amplitudes, revela que os examinadores humanos, identificados como Examinador 1 e Examinador 2, apresentaram uma diferença média absoluta (MAD) de 0.0009, indicando uma diferença média muito pequena em relação aos valores esperados. O erro percentual médio absoluto (MAPE) para ambos foi de 0.0012%, refletindo uma alta precisão nas medições, apesar de não serem absolutamente perfeitas. Em contraste, o Examinador 3, que representa o sistema de marcação automática do STEP, demonstrou uma precisão absoluta, com uma MAD e um MAPE de 0.0000, indicando que as medições automáticas estavam exatamente alinhadas com os valores esperados. Além disso, a correlação de Pearson foi quase perfeita para os examinadores 1 e 2, quando comparados aos resultados esperados, valor de *r* igual a 0.999. A correlação foi perfeita, quando os valores esperados foram comparados aos valores marcados automaticamente pelo aplicativo STEP, com valor de *r* igual 1. Isso sugere que, embora os examinadores humanos tenham apresentado pequenas variações, as medições seguiram consistentemente o padrão esperado.

A análise da segunda parte dos dados, os cálculos realizados pelo STEP, a partir das marcações dos dois examinadores, e da marcação automática do sistema STEP, mostrou pequenas diferenças entre os métodos calculados com as marcações manuais e a marcação automática. Para os examinadores humanos, a MAD de foi de 0.1111, refletindo uma discreta diferença em relação aos valores esperados. O MAPE para ambos foi muito pequeno, com valor igual a 0.0094%. Contudo, mais uma vez, os cálculos realizados com a marcação automática demonstraram uma precisão maior, com uma MAD de 0.0100 e um MAPE de 0.0037%. Além disso, a correlação de Pearson seguiu o mesmo padrão da análise dos picos e foi perfeita para os cálculos realizados com a marcação automática, quando comparados aos valores teóricos esperados.

A Tabela 2, a seguir, mostra os valores dos resultados esperados e dos resultados encontrados a partir da marcação dos picos no domínio das frequências, pelos examinadores e pelo sistema automático do STEP.

**Tabela 2:** Valores esperados e encontrados pelos examinadores, utilizando o STEP, para cada medida, marcada e calculada, por frequência gerada, no domínio das frequências

Medidas	Esperado	Examinador 1	Examinador 2	Examinador 3*
500Hz FFT: Frequência (Hz)	500	502,07	502,07	500
500Hz FFT: Amplitude (uV)	10	10	10	10
1000Hz FFT: Frequência (Hz)	1000	998,96	998,96	1000
1000Hz FFT: Amplitude (uV)	10	10	10	10
2000Hz FFT: Frequência (Hz)	2000	1997,93	1997,93	2000
2000Hz FFT: Amplitude (uV)	10	10	10	10
Soma FFT: pico 1 Frequência (Hz)	500	502,07	502,07	500
Soma FFT: pico 1 Amplitude (uV)	10	10	10	10
Soma FFT: pico 2 Frequência (Hz)	1000	998,96	998,96	1000
Sum FFT: pico 2 Amplitude (uV)	10	10	10	10
Soma FFT: pico 3 Frequência (Hz)	2000	1997,93	1997,93	2000
Soma FFT: pico 3 Amplitude (uV)	10	10	10	10

* Examinador 3: marcação automática do STEP

**Legenda:** FFT - transformada rápida de Fourier; Hz – hertz; µV – microvolts

Um exemplo dos gráficos e suas respectivas marcações no domínio das frequências, para a onda composta pelas três senoides, pode ser encontrado da Figura 3, a seguir.

**Figura 3:** Tela de marcação dos picos de frequência do aplicativo STEP. Exemplo de picos marcados para a onda somatória (0.5, 1 e 2 kHz)

A análise dos valores encontrados na FFT, com os picos marcados manualmente e com os picos marcados automaticamente, quando comparados aos valores teóricos esperados, revela diferenças discretas entre os métodos de medição manual e automático. Os examinadores humanos apresentaram uma MAD de 1.04 e um MAPE de 0.0418%, o que sugere uma variação percentual média extremamente pequena nas medições realizadas. A comparação dos resultados teóricos com os resultados calculados a partir das marcações automáticas do sistema STEP demonstrou uma precisão absoluta, com uma MAD e um MAPE iguais a 0, evidenciando que as medições automáticas estavam perfeitamente alinhadas com os valores esperados. Além disso, a correlação de Pearson foi alta para todos os examinadores, com valores iguais a aproximadamente 1 e 1, respectivamente, indicando uma correspondência perfeita entre as medições e os valores esperados.

Finalmente foi realizada uma análise adicional para comparar os resultados da área entre os limites calculados com o método da integral e pelo método da soma de dois triângulos. A análise de normalidade indicou que os dados não seguem uma distribuição normal, justificando o uso de testes não paramétricos. Foi encontrada uma diferença significativa, já esperada, entre as áreas calculadas pelo método da integral e pelo método dos triângulos, conforme indicado pelo teste de Wilcoxon, que resultou em um valor de p menor que 0.001. Esse resultado sugere que os dois métodos produzem valores diferentes para a área sob a curva. Além disso, o tamanho do efeito (*d* de *Cohen*) foi calculado para quantificar a magnitude da diferença entre os métodos, resultando em um valor de 1.68, o que indica uma grande diferença entre os métodos de cálculo das áreas.

### Etapa II

Os dados foram marcados de forma independente, pelos dois examinadores com cada um dos três sistemas, e os resultados médios e respectivos intervalos de confiança podem ser encontrados na Tabela 3, a seguir.

**Tabela 3:** Valores encontrados para latências e amplitudes do potencial evocado auditivo de tronco encefálico, eliciado por clique, por sistema e por examinador

Examinador 1	AEP			Excel			STEP		
	Onda I	Onda III	Onda V	Onda I	Onda III	Onda V	Onda I	Onda III	Onda V
**Latência**									
Média (ms)	1,45	3,55	5,33	1,43	3,57	5,3	1,42	3,53	5,29
IC 95% Limt. Inf.	1,41	3,47	5,19	1,39	3,48	5,18	1,38	3,45	5,16
IC 95% Limt. Sup.	1,49	3,63	5,47	1,47	3,66	5,42	1,46	3,61	5,42
**Amplitude**									
Média (uV)	0,17	0,23	0,16	0,15	0,21	0,15	0,17	0,22	0,16
IC 95% Limt. Inf.	0,11	0,16	0,11	0,07	0,12	0,07	0,08	0,12	0,08
IC 95% Limt. Sup.	0,23	0,3	0,21	0,23	0,3	0,23	0,26	0,32	0,24
Examinador 2									
**Latência**									
Média (ms)	1,45	3,57	5,31	1,44	3,55	5,31	1,41	3,47	5,27
IC 95% Limt. Inf.	1,41	3,49	5,17	1,4	3,45	5,19	1,37	3,37	5,14
IC 95% Limt. Sup.	1,49	3,65	5,45	1,48	3,65	5,43	1,45	3,57	5,4
**Amplitude**									
Média (uV)	0,16	0,23	0,16	0,17	0,24	0,15	0,17	0,23	0,17
IC 95% Limt. Inf.	0,1	0,15	0,11	0,08	0,16	0,06	0,08	0,14	0,09
IC 95% Limt. Sup.	0,22	0,31	0,21	0,26	0,32	0,24	0,26	0,32	0,25

**Legenda:** AEP - *Auditory Evoked Potential;* STEP - *Smart Tools for Evoked Potentials*; % - porcentagem; ms – milissegundos; µV – microvolts; IC – intervalo de confiança

O teste ANOVA não revelou nenhuma diferença significativa entre os três sistemas avaliados para as marcações das ondas do PEATE. Os valores encontrados para as latências das ondas I, III e V foram, respectivamente, F (2, 57) = 1.127 e p = 0.329, F = 1.154 e p = 0.320, e F = 0.161 e p = 0.852. Para as amplitudes os valores respectivos foram: F (2, 57) = 0.005 e p = 0.995, F = 0.013 e p = 0.987, e F = 0.129, p = 0.879.

Também não foram encontradas diferenças significativas entre os dois examinadores. Os resultados dos valores de t e p para a comparação entre as latências das ondas I, III e V, marcadas com o sistema AEP, foram, respectivamente, t = -1.00 e p = 0. 334, t = -1.468 e p = 0.164, t = 1.00 e p = 0.334. Para o sistema MS Excel os valores das latências, respectivamente, foram: t = 1.00 e p = 0. 334, t = -1.00 e p = 0. 334, t = -1.468 e p = 0.334. Para o STEP os valores das latências das ondas foram, respectivamente: t = 0.823 e p = 0. 424, t = -0.564 e p = 0. 582, t = -1.871 e p = 0.082. Para as amplitudes, os valores para cada uma das três ondas marcadas com o AEP, depois com MS Excel e, finalmente com STEP foram, respectivamente, AEP: t = 1.00 e p = 0. 334, t = -1.00 e p = 0. 334, t = -1.00 e p = 0.334, MS Excel: -1.468 e p = 0.164, t = 0.823 e p = 0. 424, t = -0.564 e p = 0. 582, e STEP: t = 1.00 e p = 0. 334, t = -1.00 e p = 0. 334, e t = -1.00 e p = 0. 334.

Na Tabela 4, podem ser encontrados os valores médios e intervalos de confiança das latências e das amplitudes para as marcações das ondas, por software e por examinador, para a marcação do PEAC.

**Tabela 4:** Valores encontrados para latências e amplitudes do potencial evocado auditivo cortical, eliciado por estímulos de fala, por sistema e por examinador

Examinador 1	*AEP*			*Excel*			*STEP*		
	P1	N1	P2	P1	N1	P2	P1	N1	P2
**Latência**									
Média (ms)	53,27	101,17	146,83	51,85	95,91	145,73	52,01	95,42	143,65
IC 95% Limt. Inf.	50,75	96,39	141,57	50,2	90,71	140,64	49,98	90,47	138,14
IC 95% Limt. Sup.	55,79	105,95	152,09	53,5	101,11	150,82	54,04	100,37	149,16
**Amplitude**									
Média (uV)	2,19	-2,89	1,62	2,09	-2,65	1,82	2,11	-2,6	1,72
CI 95% Limt. Inf.	1,41	-3,74	0,91	1,31	-3,33	1,13	1,36	-3,33	1,04
CI 95% Limt. Sup.	2,97	-2,04	2,33	2,87	-1,97	2,51	2,86	-1,87	2,4
Examinador 2									
**Latência**									
Média (ms)	53,83	99,78	148,63	52,44	96,58	147,11	52,17	98,51	146,57
IC 95% Limt. Inf.	49,84	94,67	143,48	48,41	91,46	141,85	48,09	93,23	141,58
IC 95% Limt. Sup.	57,82	104,89	153,78	56,47	101,7	152,37	56,25	103,79	151,56
**Amplitude**									
Média (uV)	2,28	-2,97	1,66	2,19	-2,59	1,75	2,32	-2,45	1,68
CI 95% Limt. Inf.	1,52	-3,77	0,96	1,42	-3,29	1,04	1,61	-3,11	0,99
CI 95% Limt. Sup.	3,04	-2,17	2,36	2,96	-1,89	2,46	3,03	-1,79	2,37

**Legenda:** AEP - *Auditory Evoked Potential;* STEP - *Smart Tools for Evoked Potentials*; % - porcentagem; ms – milissegundos; µV – microvolts; CI – intervalo de confiança

Mais uma vez o teste ANOVA não revelou nenhuma diferença significativa entre os três sistemas avaliados para as marcações das ondas do PEAC. Os valores encontrados para as latências das ondas P1, N1 e P2 foram, respectivamente, F (2, 28) = 0.528 e p = 0.594, F = 1.575 e p = 0.219, e F = 0.357 e p = 0.702. Para as amplitudes os valores respectivos foram: F (2, 28) = 0.018 e p = 0.982, F = 0.165 e p = 0.849, e F = 0.080, p = 0.923.

Também não foram encontradas diferenças significativas entre os dois examinadores. Os resultados para os valores de t e p para a comparação entre as latências das ondas P1, N1 e P2, marcadas com o sistema AEP, foram, respectivamente, t = -0.332 e p = 0. 745, t = 1.255 e p = 0.230, t = -1.22 e p = 0.242. Para o sistema MS Excel os valores das latências, respectivamente, foram: t = -0.320 e p = 0.754, t = -2.09 e p = 0.055, t = -1.692 e p = 0.113. Para o STEP os valores das latências das ondas foram, respectivamente: t = -0.093 e p = 0. 928, t = -1.798 e p = 0. 094, t = -2.031 e p = 0.062. Para as amplitudes os valores para cada uma das três ondas marcadas com o AEP, depois com MS Excel e, finalmente com STEP foram, respectivamente, AEP: t = -2.064 e p = 0. 058, t = 1.319 e p = 0. 208, t = -1.382 e p = 0.189, MS Excel: -1.118 e p = 0.282, t = -1.581 e p = 0. 136, t = 1.661 e p = 0. 119, e STEP: t = -1.520 e p = 0.151, t = -1.605 e p = 0. 131, e t = 0.505 e p = 0. 621.

## DISCUSSÃO

### Etapa I

As pequenas diferenças observadas entre os valores esperados e os valores medidos pelos examinadores podem ser atribuídas a limitações no processo de amostragem. Alguns trabalhos destacam que a precisão das medições pode ser significativamente influenciada pela taxa de amostragem utilizada. A técnica de amostragem equivalente pode ser uma solução eficiente para melhorar a precisão sem exigir taxas de amostragem extremamente altas, o que é corroborado por trabalhos que discutem a eficácia dessa abordagem em situações com alta interferência harmônica^(8,9)^.

Embora taxas de amostragem mais altas possam potencialmente reduzir os erros, elas aumentam a complexidade e os requisitos de processamento do sistema, o que pode não ser viável para todos os equipamentos e aplicações, especialmente em sistemas com limitações de processamento^(10-12)^. Assim, as taxas de amostragem atualmente utilizadas nos sistemas padrão são consideradas suficientes para garantir medições precisas na maioria das aplicações, e o aumento dessas taxas deve ser cuidadosamente considerado para evitar sobrecarga de processamento e problemas de estabilidade.

As diferenças mínimas encontradas entre os resultados esperados e os obtidos pelo software STEP (tanto nas medições manuais quanto nas automáticas) validam a utilização deste sistema para análise de sinais eletrofisiológicos. Estudos mostram que variações pequenas nas medições são comuns devido a limitações inerentes ao processo de amostragem e características do equipamento utilizado^(8)^. Mesmo com tecnologias avançadas como o *imaging* de cálcio e a eletrofisiologia, diferenças de medição podem surgir devido às variabilidades nos métodos de aquisição e processamento de dados^(13)^.

Além disso, a prática clínica rotineira de comparar resultados de exames eletrofisiológicos com padrões adquiridos no mesmo equipamento e sistema de análise ajuda a mitigar os impactos dessas variações. Diferentes equipamentos, mesmo quando conectados a uma mesma pessoa, em um mesmo momento, com o mesmo protocolo e no mesmo ambiente, podem produzir traçados eletrofisiológicos distintos devido a diferenças na calibração e nos algoritmos de processamento de sinais^(8)^. Portanto, as pequenas variações observadas nas medições do STEP, ainda que fossem maiores quando comparadas aos resultados esperados, tenderiam a afetar uniformemente o padrão e o exame analisado, permitindo comparações válidas.

A utilização do método da soma de dois triângulos^2^, ou de um triângulo^3^, ou de um retângulo^4 (14)^, ou ainda do deslocamento do pico da onda V para o zero e cálculo do retângulo, formado pela soma das amplitudes multiplicadas pelo intervalo de tempo entre os pontos da amostragem, começando no pico da onda V e terminando no vale da onda A^(15)^, podem ser encontrados para calcular a área sob a curva, entre o pico da onda V e o vale da onda A, no exame eletrofisiológico de *frequency following response* (FFR) e em outras medidas.

No entanto, mesmo o método mais aproximado, a soma dos dois triângulos, não é ideal para traçados senoidais precisos, muito menos para dados eletrofisiológicos irregulares. De acordo com a literatura, métodos de integração, como o uso de integrais definidas, são preferíveis para calcular a área com maior precisão, pois capturam melhor as variações na forma da onda^(16)^. O uso do método dos dois triângulos, ou de retângulos, pode resultar em uma subestimação ou superestimação da área real, especialmente em curvas com flutuações significativas. Isso é corroborado por estudos que mostram que métodos que aproximam a área utilizando formas geométricas simples frequentemente apresentam erros^(17)^, por isso a aproximação por dois triângulos não é a melhor opção. Métodos eficazes podem utilizar combinações de formas geométricas para calcular a *area-under-the-curve* (AUC), no intervalo entre os pontos cartesianos de uma amostra, do valor máximo até o zero e do valor mínimo até o zero, em módulos e somados, para aumentar a precisão da medida^(17)^.

### Etapa II

Os resultados do estudo com exames de pacientes indicaram que não houve diferenças significativas nas latências e amplitudes das ondas do PEATE e PEAC marcadas por dois examinadores utilizando-se três diferentes sistemas, um padrão ouro clínico (AEP), outro padrão ouro utilizado em análises matemáticas (*MS Excel*) e o novo aplicativo proposto (STEP). Também não foram encontradas diferenças significativas nas referidas marcações entre os examinadores.

Podem ser encontrados muitos estudos que referem a existência de pequenas variações nas amplitudes e latências entre os diferentes equipamentos e sistemas utilizados para a aquisição de potenciais evocados auditivos^(8)^ e devido aos diferentes protocolos utilizados^(18)^. O presente estudo, entretanto, utilizou, nesta etapa, exames realizados no mesmo equipamento e com o mesmo protocolo, cujas ondas foram marcadas em três sistemas diferentes. Dessa forma, o fato de não serem encontradas diferenças entre tais marcações indica que o aplicativo STEP pode ser utilizado clinicamente ou em pesquisas, pois possui resultados semelhantes aos dois sistemas padrão ouro testados.

Assim, caso fossem encontradas diferenças entre os examinadores, estas seriam apenas um indicativo de que não havia uma padronização da técnica utilizada por eles para marcar os exames. A consistência entre diferentes examinadores ao medir latências e amplitudes de ondas PEATE e PEAC é crucial para a confiabilidade dos dados^(19)^. O presente estudo não encontrou diferenças significativas entre os examinadores, o que está de acordo com a literatura, que aponta para a confiabilidade das medições dos potenciais evocados auditivos quando realizadas por diferentes profissionais treinados​ com a mesma técnica​. Este é um ponto positivo, pois indica que o treinamento adequado dos examinadores pode minimizar a variabilidade nas medições^(20)^, garantindo a consistência dos resultados independentemente de quem realiza as marcações.

Por fim, podem ser destacadas duas limitações do estudo e as ações realizadas para minimizá-los: 1. O uso de sinais simulados pode não capturar toda a complexidade dos sinais eletrofisiológicos reais. Para reduzir essa limitação, os parâmetros das ondas geradas foram cuidadosamente definidos para refletir as condições ideais de medição, e a precisão foi comparada com cálculos teóricos rigorosos. Além disso, posteriormente foram analisados dados reais. 2. Ao validar medidas automáticas e manuais, há sempre o risco de discrepâncias devido a erros humanos ou limitações dos algoritmos do aplicativo. Para minimizar essa limitação, o estudo incluiu revisões por matemáticos e profissionais da computação extremamente qualificados, e a comparação de resultados entre dois examinadores experientes, além da utilização de uma terceira medição automática, o que aumenta a confiabilidade dos dados.

## CONCLUSÃO

O STEP apresentou uma alta precisão para a marcação dos picos e vales e para a realização dos cálculos do slope e da área abaixo da curva entre dois limites, tanto por meio da integral como pela aproximação pela área de dois triângulos, na comparação dos resultados teóricos com os resultados experimentais. Nas análises de exames reais não existem diferenças significativas entre as latências e amplitudes das ondas do PEATE e do PEAC marcadas com o aplicativo STEP, quando comparadas com aplicativos padrão ouro, amplamente utilizados, e disponíveis no mercado.

## Footnotes

1 Todos os cálculos foram revisados por um matemático.

2

$\begin{array}{l} \left[\frac{\left(\left|amplitude_{V}\right|\right)*\left(cruza-zero_{latência}-V_{latência}\right)}{2}\right]+\\ \left[\frac{\left(\left|amplitude_{A}\right|\right)*\left(A_{latência}-cruza-zero_{latência}\right)}{2}\right] \end{array}$

3

$\left(\left|amplitude_{V}\right|+\left|amplitude_{A}\right|\right)*\left(Latência_{A}-Latência_{V}\right)$

4

$\frac{\left(\left|amplitude_{V}\right|+\left|amplitude_{A}\right|\right)*\left(Latência_{A}-Latência_{V}\right)}{2}$

## Apêndice I

Disponibilidade do software

As informações sobre o software do pacote de modelagem são as seguintes:

1. Nome do software: STEP wave generator.

2. Contato do desenvolvedor: Pedro de Lemos Menezes pedro.menezes@uncisal.edu.br.

3. Endereço: Rua Doutor Jorge de Lima, 113. Trapiche da Barra - CEP: 57010-300, Maceió, AL- Brasil.

4. Número de telefone: +55 (82) 3315-6701

5. Ano da primeira disponibilidade: 2024.

6. Hardware sugerido: Processador *Intel Core* i7 de 2,6 GHz, com 16 GB 1600 MHz DDR3 e HD de 1 TB. No entanto, realizamos as simulações em um computador Apple M1, CPU de 8 núcleos, GPU de 8 núcleos, *Neural Engine* de 16 núcleos. Sistema operacional macOS *Sonoma* 14.0.

7. *Software* necessário: Todo o projeto está sendo executado no Ubuntu 16.04 (recomendado)

8. Disponibilidade: Disponível no Github3: https://github.com/pedrolemosmenezes/Smart-Tools-Evoked-Potentials/tree/ac1b231a46d3b0fa90491d58a5d5bc1bd3ec756d

9. Custo: Todas as ferramentas gratuitas.
